# Contrast-Induced Encephalopathy Presenting With Fever After Coronary Angiography: A Case Report

**DOI:** 10.7759/cureus.32271

**Published:** 2022-12-06

**Authors:** Ali Almasood, Abdulrahman Alsenani, Almathna Alawlah, Abdulaziz Alsenani

**Affiliations:** 1 Cardiology, Specialized Medical Center, Riyadh, SAU; 2 Medicine, Majmaah University, Majmaah, SAU; 3 Internal Medicine, Specialized Medical Center, Riyadh, SAU

**Keywords:** iodinated contrast agents, contrast-induced encephalopathy, adverse effects, reversible encephalopathy, cardiac catheterization complications, coronary angiography, contrast media, contrast-induced neurotoxicity

## Abstract

Contrast-induced encephalopathy (CIE) is a rare complication that occurs after exposure to contrast media, and it usually manifests with transient neurological deficits that include cortical blindness, altered mental status, and paralysis. It is self-limited, and symptoms usually resolve within 48-72 hours. It requires a high index of suspicion and must be taken into consideration in every patient developing a neurological manifestation after the administration of radiocontrast media. We report a case of post-coronary angiography contrast-induced encephalopathy with low-grade fever and negative imaging.

## Introduction

The use of contrast agents to aid the diagnosis of patients has a low prevalent risk. The side effects of radiological contrast administration are either idiosyncratic, like angioedema and bronchospasm, or organ-specific, like acute renal failure. One of these side effects relating to the central nervous system is contrast-induced encephalopathy (CIE) [1].

CIE is a rare complication that occurs after exposure to contrast media [2], and it usually manifests with transient neurological deficits that include cortical blindness, altered mental status, and paralysis [3]. It is self-limited, and symptoms usually resolve within 48-72 hours, but there have been documented cases of fatal cerebral edema and death secondary to CIE associated with the use of ionic high-osmolar contrast agents [4].

Although the mechanism of CIE remains unclear, it is hypothesized to be due to osmotic disruption of the blood brain barrier (BBB). Hypertension, diabetes mellitus, renal impairment, and the administration of large volumes of iodinated contrast are common risk factors for developing CIE [5].

Different kinds of contrast media, which include ionic, non-ionic, hyperosmolar, and iso-osmolar, as well as different doses, have all been reported to induce CIE [2]. We report a case of post-coronary angiography contrast-induced neurotoxicity with low-grade fever and negative imaging.

## Case presentation

**Figure 1:**
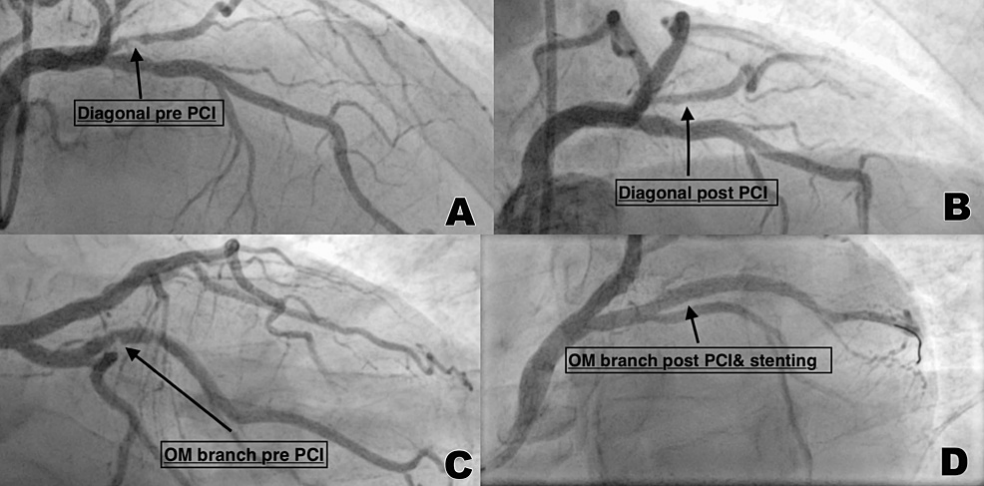
Left-Sided Heart Angiogram (A) Pre-interventional critical stenosis of the ostium of the first diagonal coronary artery. (B) Post-interventional patency of the ostium of the first diagonal coronary artery after balloon angioplasty with a drug-eluting balloon. (C) Pre-interventional severe stenosis of the first obtuse marginal coronary artery. (D) Post-interventional patency of the first obtuse marginal coronary artery after angioplasty and stenting. PCI: percutaneous coronary intervention; OM: obtuse marginal

A 68-year-old male with a history of hypertension, type-II diabetes mellitus, and dyslipidemia controlled medically, presented with worsening chest pain for one month and was admitted to the cardiology unit for coronary angiography and possible percutaneous coronary intervention. The patient was afebrile and vitally stable on admission. Physical examination, including neurological examination, was unremarkable. A diagnostic coronary angiogram was performed through the right radial access with a six-french catheter, which revealed double vessel disease with critical stenosis of the ostium of the first diagonal coronary artery as depicted in Figure 1A, and severe stenosis of the first obtuse marginal coronary artery as depicted in Figure 1C. Balloon angioplasty with a drug-eluting balloon was performed successfully on the ostium of the first diagonal coronary artery (Figure 1B). Angioplasty was performed, and one drug-eluting stent was implanted in the first obtuse marginal coronary artery (Figure 1D). A total of 190 ml of Omnipaque (Iohexol), a low osmolality, non-ionic, iodine-based contrast agent, was administered during the procedure.

In the catheterization laboratory, following our periprocedural protocol, the patient was given lidocaine 1% as a local anesthetic. Also, verapamil 200 mcg and nitroglycerin 200 mcg were administered intra-arterially as a radial cocktail. The patient received 1 mg of midazolam and 25 mcg of fentanyl. Three doses of 5,000 IU heparin were administered throughout the procedure, with a final activated clotting time of 243 seconds and no intraprocedural complications.

**Figure 2:**
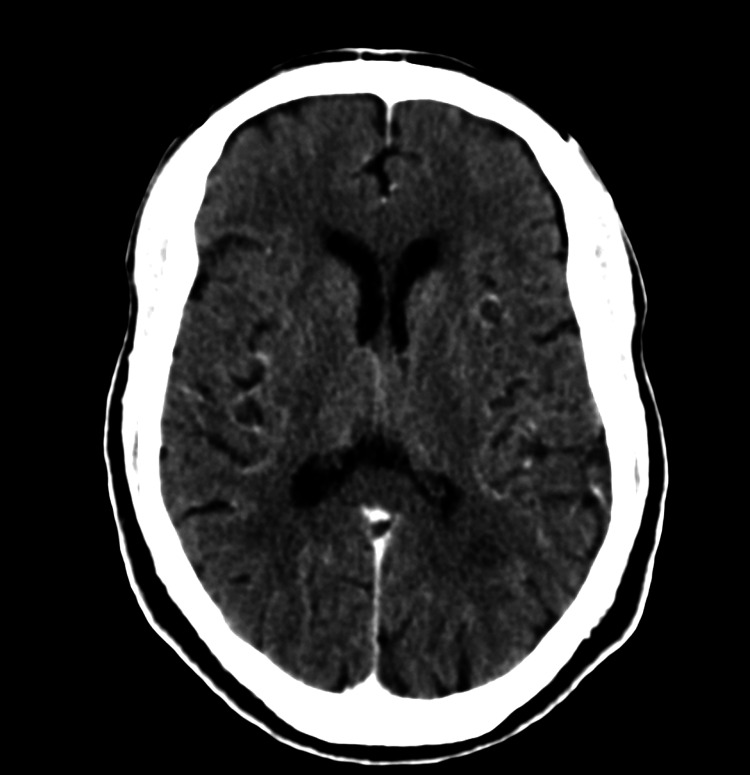
Non-contrast CT scan of the head one hour after coronary angiography

Immediately after the procedure, the patient became less cooperative and his mental status was progressively worsening. Forty-five minutes after the procedure, the patient became agitated and aggressive. Upon neurological examination, he was confused and disoriented to person, time, and place, with bilateral 5/5 power and +2 reflexes. He was started on aggressive intravenous (IV) hydration and received 10 mg haloperidol and emergency non-contrast head computed tomography (CT) was performed within 1 hour, which showed no evidence of any acute pathological findings (Figure 2). Brain magnetic resonance imaging (MRI), was planned and as the patient remained symptomatic, a dose of 25 mg of quetiapine was administered. New investigations were ordered, including electrolyte and kidney function tests, which both were unremarkable. 

Eight hours after the procedure, the patient developed a fever of 38.8 C for which a full septic workup was done, no source of infection was identified, and all inflammatory markers (erythrocyte sedimentation rate (ESR), C-reactive protein (CRP), procalcitonin) were negative. A brain MRI performed 36 hours after the procedure showed no acute pathological findings. The patient’s symptoms were managed with aggressive fluid hydration and midazolam when needed, reaching a collective dose of 16 mg over a course of 48 hours. Within those 48 hours of supportive management, the patient's fever subsided and his clinical status started to improve, ultimately making a full recovery to baseline with no residual neurological deficits.

## Discussion

CIE is an extremely rare complication associated with the administration of intravascular radiocontrast media, which seems to affect subjects receiving localized contrast medium injection [2], with only two documented exceptions [4,6].

The first documented case of CIE, which manifested as transient cortical blindness after coronary angiography was reported in 1970 [2,7]. The total number of documented cases of CIE after coronary angiography was 75, based on a systematic review published in December 2020 [3]. The exact mechanism of CIE is unknown, but studies have shown that a combination of disruption in the BBB and the direct neurotoxic effect of the contrast are the cause of such a presentation, in which the hyperosmolality of the contrast causes shrinking in the cells of the BBB, such disruption causes the toxic contrast to cross to the CNS [8,9]. Other studies have shown that some factors other than the hyperosmolarity of the media may cause disruption of the BBB, one of which is the contrast-induced release of endothelin causing increased BBB permeability [10]. The clinical presentation of CIE varies greatly, the commonest symptoms include cortical blindness (58%), altered mental status (24%), headache (7%), limb paralysis or weakness (7%), and seizures (5%). Our patient did not develop cortical blindness, but an altered mental status was the primary complaint. There is great variability in severity from one patient to another, but the type of contrast used as well as the dose administered seems to have no correlation with the severity or acuteness of symptoms [3].

Fever is a rare manifestation of CIE that has not been very well-documented in the literature, with only a few cases that showcased fever in the context of CIE. Most cases that presented with fever were low-grade, and most of the cases did not mention the fact that fever of an underlying cause was excluded [11,12]. In this case, a full septic workup was done to rule out other possible differential diagnoses like meningitis, encephalitis, and abscesses but no focus of infection was identified. The mechanism behind the fever could be explained by the contrast media acting directly on the hypothalamus and causing a stimulatory effect that alters thermoregulation [9].

In this case, the patient developed symptoms within one hour, which is consistent with other studies showing that the onset of symptoms occurs within minutes to hours and up to days from the procedure. With regard to the resolution of symptoms, the majority of cases show resolution of the neurological deficits within 48-72 hours [3]. Our case was in concordance with those findings, having a spontaneous resolution of the symptoms occurring within 48 hours.

The diagnosis of CIE is largely based on excluding other feared sequelae of coronary angiography like thromboembolic complications, hemorrhage, and masses using brain CT and MRI. The brain CT of our case, performed immediately after the onset of symptoms, showed no acute pathological findings, but initial CT scans in patients with CIE may either be normal or demonstrate a variety of pathological signs like cerebral edema, cortical or subcortical enhancement, or other findings that mimic subarachnoid hemorrhage or intracerebral hemorrhage [13].

Regarding the risk factors that have been associated with CIE, one meta-analysis showed that higher contrast doses (OR, 1.072; CI, 0.952-1.192), and having a history of stroke (OR, 5.153; CI, 1.726-8.581), were significant risk factors for developing CIE. Other significant risk factors were renal dysfunction, anticoagulant use, heart failure, Ioversol (as compared with iopromide), and isolated posterior circulation cerebral angiography. They have also found that visual manifestations were more common in patients that were given a contrast dose of more than 150 mL (OR, 7.083; CI, 1.172-42.793) [14]. However, one case showed manifestations after the administration of only 25 ml of iopromide with symptoms developing immediately within the procedure [15].

The majority of patients with CIE have a good prognosis and recover completely without any residual neurological deficits using only supportive therapy like aggressive IV fluids and symptomatic control [5], but that is not always the case, as there have been reports of some cases with persistent deficits, as well as autopsy-confirmed cases of fatal cerebral edema in response to the administration of ionic high osmolar contrast agents [16]. Even though supportive therapy remains the mainstay of treatment, there are some documented cases that support the use of intravenous steroids and anti-edema agents [2,4,17].

## Conclusions

CIE remains a rare complication that requires a high index of suspicion and must be taken into consideration in every patient developing a neurological manifestation after the administration of radiocontrast media. The different possible manifestations of CIE must be researched extensively to be able to rapidly identify such patients and avoid unnecessary treatment. It is of most importance to exclude post-coronary angiography complications and to rule out any underlying infection that would explain the fever with a full septic workup.
